# Abiotic stress experiments need a reality check to improve translation to the field

**DOI:** 10.1093/jxb/erac509

**Published:** 2023-01-10

**Authors:** Anne Plessis

**Affiliations:** School of Biological and Marine Sciences, Plymouth University, Drake Circus, Plymouth PL4 8AA, UK; University of Birmingham, UK

**Keywords:** Abiotic stress, crop improvement, environmental reductionism, field, reproducibility, salt stress

**Box 1. The contrast between salt stress in the field and in the lab**

In agricultural settings, salt stress occurs most commonly in fields with saline soils (Hopmans *et al.*, 2021). Crops growing in coastal areas can also encounter salt stress in the forms of brackish water irrigation, sea water flooding, saline water getting into paddy fields, and sea water spray. All of these scenarios involve the presence of a soil matrix (even if saturated with water in the case of paddy fields and flooding), yet many salt stress studies are conducted in agar media or using hydroponic systems. Inland forms of salt stress develop gradually and slowly, as ions that were drained through the soil come closer to the surface with evaporation during the life of the plant (Shavrukov, 2013). In contrast, many studies apply a high concentration of salt suddenly, causing an osmotic shock (Shavrukov, 2013) that only some coastal plants would have evolved to face. Even the incremental increases in salt concentration being used in some experiments are not representative of a plant starting its life in saline soil. Furthermore, when a soil matrix is used for the experiment, the salt is usually added in a homogeneous manner, while ion concentrations in saline soils are highly heterogeneous (Valenzuela *et al.*, 2022). While these are all major changes to the form of saline stress the plants will experience in the field, the most overlooked and unjustifiable reductionist aspect of many salt stress experiments is in the composition of the salt. In sea water, NaCl forms ~86% of the weight of all ions and the salt composition of saline soils ranged from 50% to 80% of NaCl (Northcote and Srene, 1972); the other main ions are sulfate, calcium, magnesium, potassium, bicarbonate, and carbonic acid. Despite this, the overwhelming majority of salt stress experiments use NaCl only, ignoring the impact of other physiologically important ions.

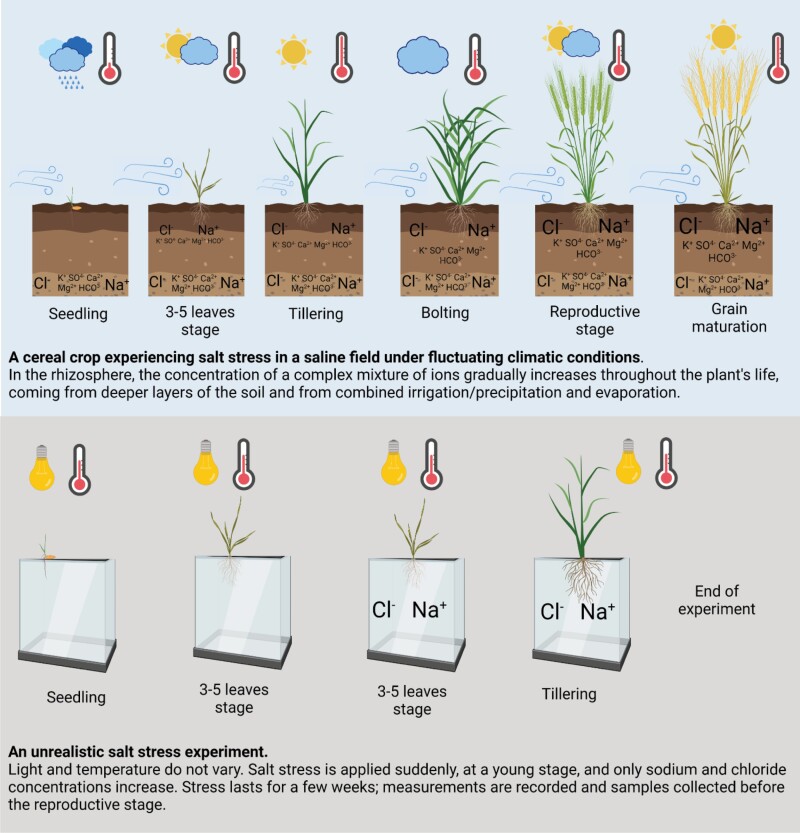

**In nearly every issue of this journal and other general plant biology journals, you will find articles on plant abiotic stress responses and signalling. These publications justify a focus on a particular stress acclimation mechanism by reviewing impacts of the associated stress on crop yield. Since the advent of genomics, the focus of abiotic stress response studies has shifted from whole-plant physiology to cellular and molecular mechanisms. Yet, little of this knowledge has been translated into improved crops that can better withstand abiotic constraints, lagging far behind the development of varieties resistant to pests and pathogens (** **Passioura, 2020** **).**

The emphasis on cells and genes has contributed an in-depth understanding of the complex processes in play when plants experience abiotic stress, uncovering for example the many roles of abscisic acid (ABA) in stress response, its metabolism, transport, and signalling (Sah *et al.*, 2016). It is now essential that we ask ourselves what we can do to fulfil the promise of the crop genomics era to provide DNA sequences that can be selected, modified, or introduced to confer better performance to crops under abiotic stresses. Several causes of the lack of translatability of mechanistic knowledge have already been identified: trying to improve complex traits depending on many genes by acting on a single gene (Budhlakoti *et al.*, 2022) and a trade-off between stress resilience and growth under optimal conditions (Krannich *et al.*, 2015). Another important issue is how we perform our abiotic stress experiments.

## The problems of environmental reductionism

While the techniques we have used to understand the genetic make-up of plants and to study the changes that occur when plants experience abiotic stress have become more and more sophisticated, we have neglected the complexity of the environment to which the plants were subjected and how it might impact what we are measuring. This issue is rooted within the very beginnings of plant physiology. From its start, the field of physiology has been influenced by principles of physics. Early plant physiologists such as Marriotte and Nicolas Sarrabat were often also physicists (Simonetta, 2003) and designed experiments with a reductionist approach (reductionism is the idea that complex processes can be understood by studying separately each of its parts), as instigated by Newton and Descartes, among others (Mazzocchi, 2008). Their legacy to this day is an oversimplification of the environment in our experiments, which could be named ‘environmental reductionism’. Many experiments are conducted in controlled-climate rooms or cabinets where light quality and intensity, temperature, and humidity stay the same through the life of our plants (except for possible day–night differences). However, it is now well established that photosynthesis works very differently under fluctuating than under steady light (Slattery *et al.*, 2018) and, given the importance of photosynthesis on abiotic stress responses, it is likely that steady light experiments provide a flawed view of abiotic stress responses in the field. The same is likely to be true of other factors such as temperature and humidity, but the effect of their fluctuations has not been researched as much. Experimental conditions are also simplified by the nearly ­complete removal of wind, even though it has an extensive effect on plant growth and physiology (Gardiner *et al.*, 2016). Convenience is another source of unrealistic experimental conditions: to ensure fast growth and healthy plant material, the typical values of light intensity and temperature in indoor experiments are well out of the range of values plants would experience in the wild (Poorter *et al.*, 2016).

Environmental reductionism has an even greater impact on how we expose our plants to the very abiotic stresses we are studying. While in nature most stresses occur gradually (with the exception of flooding), sometimes with fluctuations in intensity, often over long periods of time, we mainly apply stress in a reductionist form: sudden, acute, and for a short amount of time, generally a few days but sometimes less than a day. Environmental reductionism also results in studying single stresses rather than combinations of stresses, even though in agricultural fields concurrent stresses are very common and damaging ­(Suzuki *et al.*, 2014). The other discrepancies between how abiotic stresses are applied in experiments and how they occur in nature are too numerous to detail here, so I have chosen to focus on how high salinity is studied in simplified and unrealistic conditions (Box 1) and the consequences of this reductionism for understanding salt stress responses in the field (Box 2).

Environmental reductionism is not only a problem for fundamental studies of abiotic stress responses but also affects molecular breeding. Due to the variability of field environments, large population phenotyping is often done in controlled environments (Gilliham *et al.*, 2017), leading to the type of discrepancies described in Box 2 and possibly to the selection of polymorphisms that have little effect in the field.

## The misguided quest for reproducibility

An often mentioned justification for using unrealistically controlled experimental conditions is to produce reproducible results. For example, the solution in a hydroponics system can be simply described and recreated for a new experiment, while soils are much more difficult to fully characterize. However, if we all use somewhat similar experimental conditions, does that make the results of our studies more reliable? This issue of environmental standardization was already discussed over a decade ago in the biomedical community. One team demonstrated that, for laboratory mice, environmental standardization impaired, rather than helped, reproducibility, with micro-differences between apparently similar settings being to blame for conflicting results (Richter *et al.*, 2009). Our model system is also not immune to microenvironmental effects: Arabidopsis plants grown in the same (or as similar as possible) conditions in nine different labs showed significant differences in growth and gene expression, in some cases to an extent comparable with the effect of some abiotic stress treatments (Massonnet *et al.*, 2010).

To improve translation to the field, it is more important to check that our results hold true across a range of environmental conditions, rather than being reproducible for one instance of stress in one specific environment, so we should switch to studying dose responses (testing many different levels of stress intensity) rather than stress versus unstressed controls (Poorter *et al.*, 2016). It is likely that some aspects of abiotic stress response considered common to many species are actually artefacts due to growing plants under stable conditions, without wind or being exposed to an impossibly fast appearing stress, and have no relevance to the field.

## Recommendations

We may be getting close to the limit of what can be learned from ‘simple’ experiments, which would mean that to gain more reliable and translatable knowledge, we must accompany advances in the technologies used to decipher biological processes with more sophisticated experimental conditions. The consequences of environmental reductionism and standardization in plant abiotic stress studies need to be much more fully investigated so that more realistic, and translatable, experiments can be designed. In addition to the questions developed in Boxes 1 and 2, here are examples of questions to address: when carrying out heat stress experiments, is moving field-grown plants to growth chambers for the stress treatment a good proxy for a real stress? How do climatic fluctuations and wind affect how plants experience and respond to abiotic stresses?

It would also be useful to fund and provide easy access to ‘field validation’ facilities, with rainout shelters for drought experiments, saline soil fields, field heaters/coolers, and field-trained technicians, where researchers could test their mutants and genetically modified lines and check whether phenotypes observed in controlled conditions hold true in the field.

In the meantime, all of us should question our experimental designs and learn the characteristics of our favourite abiotic stress in the field (which might depend on the climate and species of interest): at what stage(s) of plant development does it usually occur? How long does it last? Are there any warning signs of the stress that the plant can detect? Are there any other environmental factors that might change during the stress (e.g. high temperature, high light intensity, and low humidity during droughts)? This should not only influence our experimental designs but also allow us to better understand the limitations of our experiments to not overstretch our conclusions. Like Richter *et al.* (2009), I also recommend introducing environmental heterogeneity in experimental designs to achieve more generalizable results.

The last decades’ emphasis on molecular biology has provided invaluable information on the mechanisms regulating plant abiotic stress responses, but has also eclipsed the environmental aspects of abiotic stress studies. Putting the spotlight back on the environment might help us uncover not only more productive information on which genes to select or modify to improve crop resilience, but also new ways to increase crop ­resilience to abiotic stress that rely on agricultural management rather than genetic improvement. This type of solution might actually take less time to implement than breeding or genetic modification, which is essential given how quickly climate change is affecting food production.

**Box 2. Known consequences of reductionist approaches to studying salt stress**

In most cases, the impact of the discrepancies between experimental and field versions of salt stress on the validity of the results obtained is poorly understood; however, the few times this has been investigated, the results are eye opening. The level of tolerance and acclimation mechanisms of barley have been shown to vary greatly between soil and hydroponics systems, even leading to a negative correlation between grain yield in the field and salt tolerance in hydroponics across 15 genotypes (Tavakkoli *et al.*, 2012). Homogeneous salt concentrations in the soil impair our ability to appropriately study ion exclusion, water uptake, and root plasticity in saline soils, among other things (Valenzuela *et al.*, 2022). The direct application of high concentrations of salt induces an osmotic shock, which is likely to involve plasmolysis of root cells and abundant electrolyte leakage, resulting in the expression of very different genes from those during gradually applied salt stress (Shavrukov, 2013). As for salt composition, the ‘minor’ ions accumulating beside sodium and chloride in saline soils should not be ignored as they modulate the physiological effects of high NaCl concentrations. For example, one of the main phytotoxicological impacts of sodium is the disruption of potassium homeostasis, yet this can be alleviated with reasonable concentrations of potassium (Kronzucker *et al.*, 2013). Few studies have compared the effect of NaCl and more realistic mixes of ions on plants, apart from recent work showing how white clover responses to NaCl solutions were markedly different (and more lethal) than exposure to acute flooding with sea water or commercial marine aquarium salt solutions (Hanley *et al.*, 2020).

## References

[CIT0001] Budhlakoti N , Kushwaha AK, Rai A, et al. 2022. Genomic selection: a tool for accelerating the efficiency of molecular breeding for development of climate-resilient crops. Frontiers in Genetics 13, 832153. 35222548 10.3389/fgene.2022.832153 PMC8864149

[CIT0002] Gardiner B , Berry P, Moulia B. 2016. Review: wind impacts on plant growth, mechanics and damage. Plant Science 245, 94–118. 26940495 10.1016/j.plantsci.2016.01.006

[CIT0003] Gilliham M , Able JA, Roy SJ. 2017. Translating knowledge about abiotic stress tolerance to breeding programmes. The Plant Journal 90, 898–917. 27987327 10.1111/tpj.13456

[CIT0004] Hanley ME , Sanders SK, Stanton H-M, Billington RA, Boden R. 2020. A pinch of salt: response of coastal grassland plants to simulated seawater inundation treatments. Annals of Botany 125, 265–276. 31329822 10.1093/aob/mcz042 PMC7442401

[CIT0005] Hopmans JW , Qureshi AS, Kisekka I, et al. 2021. Critical knowledge gaps and research priorities in global soil salinity. Advances in Agronomy 169, 1–191.

[CIT0006] Krannich CT , Maletzki L, Kurowsky C, Horn R. 2015. Network candidate genes in breeding for drought tolerant crops. International Journal of Molecular Sciences 16, 16378–16400. 26193269 10.3390/ijms160716378 PMC4519955

[CIT0007] Kronzucker HJ , Coskun D, Schulze LM, Wong JR, Britto DT. 2013. Sodium as nutrient and toxicant. Plant and Soil 369, 1–23.

[CIT0008] Massonnet C , Vile D, Fabre J, et al. 2010. Probing the reproducibility of leaf growth and molecular phenotypes: a comparison of three Arabidopsis accessions cultivated in ten laboratories. Plant Physiology 152, 2142–2157. 20200072 10.1104/pp.109.148338 PMC2850010

[CIT0009] Mazzocchi F. 2008. Complexity in biology. EMBO Reports 9, 10–14. 18174892 10.1038/sj.embor.7401147 PMC2246621

[CIT0010] Northcote KH , Srene JKM. 1972. Australian soils with saline and sodic properties. Canberra: CSIRO.

[CIT0011] Passioura JB. 2020. Translational research in agriculture. Can we do it better? Crop and Pasture Science 71, 517–528.

[CIT0012] Poorter H , Fiorani F, Pieruschka R, Wojciechowski T, van der Putten WH, Kleyer M, Schurr U, Postma J. 2016. Pampered inside, pestered outside? Differences and similarities between plants growing in controlled conditions and in the field. New Phytologist 212, 838–855. 27783423 10.1111/nph.14243

[CIT0013] Richter SH , Garner JP, Würbel H. 2009. Environmental standardization: cure or cause of poor reproducibility in animal experiments? Nature Methods 6, 257–261. 19333241 10.1038/nmeth.1312

[CIT0014] Sah SK , Reddy KR, Li J. 2016. Abscisic acid and abiotic stress tolerance in crop plants. Frontiers in Plant Science 7, 571. 27200044 10.3389/fpls.2016.00571 PMC4855980

[CIT0015] Shavrukov Y. 2013. Salt stress or salt shock: which genes are we studying? Journal of Experimental Botany 64, 119–127. 23186621 10.1093/jxb/ers316

[CIT0016] Simonetta AM. 2003. Short history of biology from the origins to the 20th Century. Florence: Firenze University Press.

[CIT0017] Slattery RA , Walker BJ, Weber APM, Ort DR. 2018. The impacts of fluctuating light on crop performance. Plant Physiology 176, 990–1003. 29192028 10.1104/pp.17.01234 PMC5813574

[CIT0018] Suzuki N , Rivero RM, Shulaev V, Blumwald E, Mittler R. 2014. Abiotic and biotic stress combinations. New Phytologist 203, 32–43. 24720847 10.1111/nph.12797

[CIT0019] Tavakkoli E , Fatehi F, Rengasamy P, McDonald GK. 2012. A comparison of hydroponic and soil-based screening methods to identify salt tolerance in the field in barley. Journal of Experimental Botany 63, 3853–3867. 22442423 10.1093/jxb/ers085 PMC3388819

[CIT0020] Valenzuela FJ , Reineke D, Leventini D, Chen CCL, Barrett-Lennard EG, Colmer TD, Dodd IC, Shabala S, Brown P, Bazihizina N. 2022. Plant responses to heterogeneous salinity: agronomic relevance and research priorities. Annals of Botany 129, 499–518. 35171228 10.1093/aob/mcac022 PMC9007098

